# Analysis and Prediction of Wear Performance of Different Topography Surface

**DOI:** 10.3390/ma13225056

**Published:** 2020-11-10

**Authors:** Ben Wang, Minli Zheng, Wei Zhang

**Affiliations:** Key Laboratory of Advanced Manufacturing and Intelligent Technology, Ministry of Education, Harbin University of Science and Technology, Harbin 150080, China; wang124133433@126.com (B.W.); minli@hrbust.edu.cn (M.Z.)

**Keywords:** surface topography, finite element simulation, surface texture roughness parameters, wear resistance, GM(0,6) model

## Abstract

Surface roughness parameters are an important factor affecting surface wear resistance, but the relevance between the wear resistance and the surface roughness parameters has not been well studied. This paper based on the finite element simulation technology, through the grey incidence analysis (GIA) method to quantitatively study the relevance between the wear amount of per unit sliding distance (Δ*V_s_*) and the surface texture roughness parameters under dry friction conditions of the different surface topography. A zeroth order six-variables grey model, GM(0,6), for prediction the wear characteristic parameter Δ*V_s_* was established, and the experiment results verified that the prediction model was accurate and reasonable.

## 1. Introduction

Tribology is a science that studies the relative movement between contact surfaces. The friction process not only consumes energy, but also generates wear and material loss [1]. The study found it is not necessarily the case that smoother surfaces have better wear resistance [2]. A proper surface texture of the contact surface can effectively improve the tribological properties of the surface [3,4]. Recently, many scholars have carried out research on the wear resistance of contact surfaces. Etsion et al. took the lead in applying lasers to the manufacturing of topographical surfaces, and studied their friction and wear properties [5]. Yu et al. found that the surface shape and orientation have significant impacts on tribological properties [6,7]. Nuraliza et al. found that a micro-pit topography is an important factor that affects the surface tribological properties [8]. Wang, Tang, and others studied circular pits in AISI1045 steel, and found that the tribological properties were better than those of non-textured surfaces [9,10].

Researchers have extensively studied the tribological properties of various different surface topographies, and clarified the positive effect of surface topography on wear resistance [11,12,13]. The surface roughness parameter is the most commonly used and direct method to characterize the surface topography, and it is very closely related to the surface wear resistance. So far, the research on the correlation between surface wear resistance and surface roughness parameter has mainly focused on the changes of tribological properties caused by a single surface roughness change [14,15,16]. The change of surface topography will cause the overall change of surface roughness parameters, and the study of a single parameter cannot accurately reflect the changing characteristics of the surface wear resistance. Therefore, it is necessary to consider the influence of surface roughness parameters on wear resistance as a whole.

Ball-end milling is the most commonly used processing method in mold manufacturing [17]. During the milling process, the surface topography was machined by the ball-end milling cutter should be a quadrangular pit structure, and Zheng et al. has studied the wear resistance of the quadrilateral pits surface topography [18]. However, in the actual processing, the cutting-in angle of the tool and the workpiece may not be exactly the same each time, different cutting-in angles will cause changes in the surface topography. Due to the coupling effect of adjacent processing paths, the ball-end milling surface topography is a unique polygonal pit structure. For the same ball-end milling processing parameters, when the cutting-in angle is different, the texture shape of the surface is also different. The main reason is that when adjacent milling paths have different cutting-in angles, the surface texture of adjacent paths will offset, and the surface topography will change from quadrilateral pit shape to polygonal pit shape under the influence of processing coupling effect between adjacent paths. In the ball-end milling process, different cutter cutting-in angles will produce different topography offset distances (*d*), forming different surface topography, and resulting in different surface wear resistance, but so far no scholar has analyzed the wear resistance of the polygonal pits.

In this study, the aim was to establish the relevance between different surface texture roughness parameters and surface wear resistance, and select suitable parameters to characterize and predict wear resistance. The research was applied to the polygonal pits surface topography of processed by ball-end milling. Therefore, this paper takes the surface with different topography offset distances of Cr12MoV hardened die steel as the research object, and a reciprocating sliding wear model of different surface topography was established using finite element simulation technology. Then, the wear characteristic parameters in the simulation were combined with the surface texture roughness parameters obtained by reverse engineering technology, and the relevance between the Δ*V_s_* and the surface texture roughness parameters was quantified analysis by the method of grey incidence analysis. Based on this, a GM(0,6) model for predicting Δ*V_s_* was established, which provides a new idea for exploring the effect of the surface roughness parameters of different surface topography with the wear performance.

## 2. Numerical Simulation of Sliding Wear Process

### 2.1. Wear Simulation Model

The Archard model is the most commonly used predictive model in the sliding wear simulation process and has been widely used in many fields [19,20]. The model shows that the wear volume is inversely proportional to the hardness of the soft material *H*, and proportional to the normal force *F* and the sliding distance *s.*

(1) $$V=k\frac{F}{H}s$$

where *k* is the wear coefficient. Combined with the basic idea of the finite element simulation, the sliding distance at node *i* is expressed as an infinitesimal form, then the Equation (1) can be expressed as [20]

(2) $$dh_{i}=\frac{k}{H}p_{i}ds_{i}$$

where *dh* is the incremental change in the surface node position and *ds* is the incremental sliding distance during the time period ∆*t*, *p_i_* is the average contact pressure.

### 2.2. Wear Simulation Procedure

**Figure 1:**
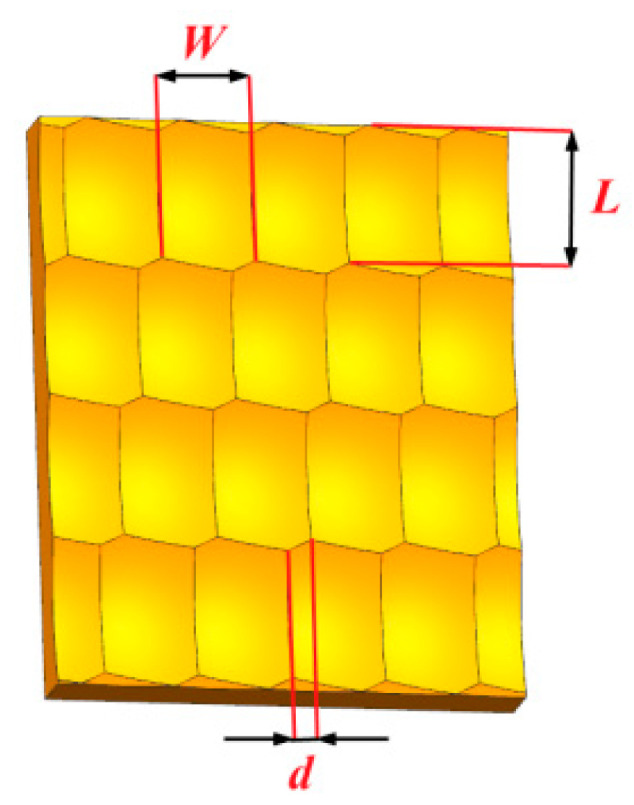
Schematic diagram of slider surface with topography offset distance.

The reciprocating sliding wear mode between the plate and the slider was used in the finite element simulation. To accurately reflect the wear characteristics between different surface topographies, the contact surface of the flat plate was an ideal plane, and the contact surface of the slider has a different surface topography. The contact surface of the slider adopts the ball-end milling surface topography model, and uses a different topography offset distance along the width direction to generate five groups of different surface topographies respectively. The topography offset distance was caused by the different initial cutting-in angles of the cutter and the workpiece during the ball-end milling machining process, and different initial cutting-in angles will produce different topography offset distances. The ball-end milling surface topography with offset distance is shown in Figure 1.

**Figure 2:**
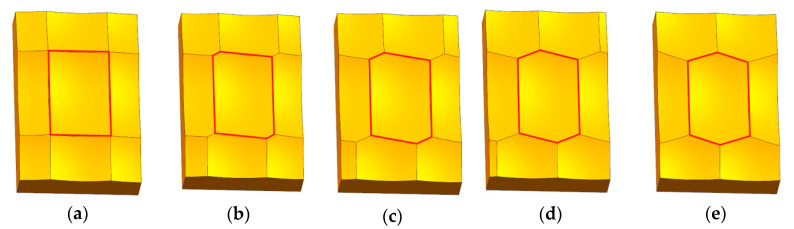
Surface topography of the five groups sliders in the simulation: (**a**) group A; (**b**) group B; (**c**) group C; (**d**) group D; (**e**) group E.

The length of the slider is 1.2 mm and the width is 0.8 mm, and the pit length (*L*) is 0.6 mm and the width (*W*) is 0.4 mm. The slider height (*h_s_*) and pit height (*h_p_*) of five groups of different offset distance surface topographies is shown in Table 1. Five group slider surface topographies in the simulation are shown in Figure 2. 

It can be seen from Figure 2 that with the increase of the topography offset distance, the surface topography of the sliders from Group A to Group E is different from each other, and gradually changes from a quadrilateral pit to an axisymmetric hexagon pit.

### 2.3. Numerical Simulation Method of Wear

In the numerical simulation of the wear process, the contact equation of the model motion process needs to be established, the wear amount was calculated by solving the equation, and then the wear offset process of the corresponding node was realized through the finite element analysis software, and complete the re-meshing of the model through the adaptive adjustment technology, so as to realize the numerical simulation analysis.

**Figure 3:**
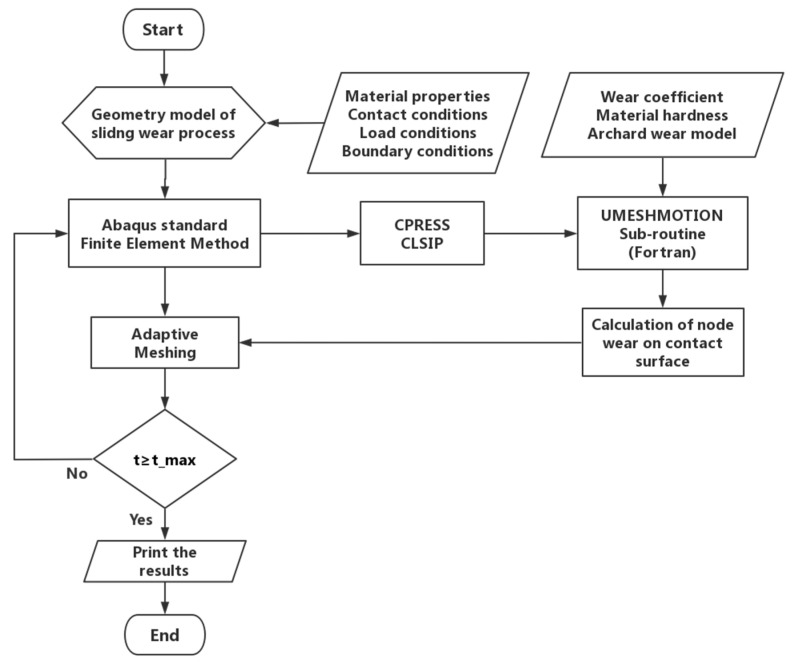
Flowchart of the finite element simulation analysis of sliding wear process.

In this paper, the ABAQUS software (ABAQUS Inc, Johnston, RI, USA) was used to complete the numerical simulation analysis. In the simulation analysis, the sliding distance CSLIP and the node normal contact pressure CPRESS were used as model variables, the parameter equation of the sliding wear process was established based on the Archard model, and uses FORTRAN language to write the Umeshmotion subroutine. Finally, ALE meshing technology was used to complete the re-meshing after the node wear. The flowchart of the sliding wear numerical simulation analysis is shown in Figure 3.

To more accurately reflect the influence of surface topography on wear, the flat plate was approximated as a rigid body, and the slider was a deformable body with different surface topography. According to the Hertz theory, there was a certain contact zone between the slider and the plate under the external normal load. With the gradual increase of the wear amount, the contact zone gradually becomes larger.

**Figure 4:**
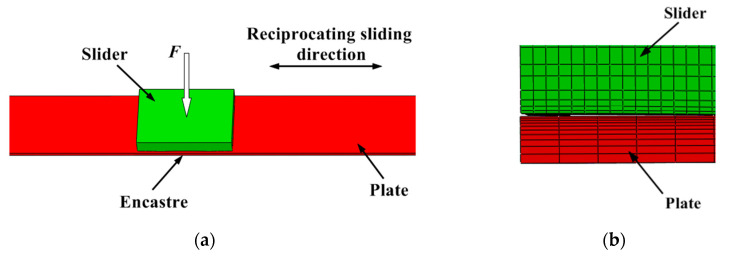
Numerical simulation model of sliding wear: (**a**) Sliding wear geometry model; (**b**) Local mesh of contact model.

In order to improve the calculation efficiency of the numerical simulation, the mesh of the contact zone was finer, and the mesh far from the contact zone was coarser. The numerical simulation model is shown in Figure 4.

This article only analyzed the relationship between wear resistance and surface topography, and the effects of temperature, wear debris, and other factors were not taken into account under dry friction conditions. The normal load of the slider is 5 MP, and it was given in the uniform pressure load manner. The average sliding speed is 150 mm/s, the frequency is 5 Hz, and the wear coefficient is 1 × 10^−5^ [21]. The materials of the flat plate and the slider are Cr12MoV hardened die steel, and their mechanical performance parameters are listed in Table 2.

## 3. Grey System Theory

Grey system theory was proposed by Deng Julong in 1982, it is a research method for ‘small data’ and ‘poor information’ [22]. This method extracts valuable information by mining partially known information, so as to realize the accurate description and analysis of system operation behavior and evolution law.

### 3.1. Grey Incidence Operator

In the grey system analysis, if the meaning and dimension between the system behavior characteristic sequence and the related factor are not the same, it needs to be non-dimensional processed through the function of the grey incidence operator to conduct subsequent analysis and research. Commonly used grey incidence operators mainly include averaging operator, interval operator, initialing operator, etc. [23]. In this paper, we choose the initialing operator to analyze the data.

Let *X_i_* = (*x_i_*(1), *x_i_*(2), …, *x_i_*(*n*)) be the behavioral sequence of factor *X_i_*. Sequence operator *D* satisfies *X_i_D* = (*x_i_*(1)*d*, *x_i_*(2)*d*, …, *x_i_*(*n*)*d*), and

(3) $$x_{i}\left(l\right)d=\frac{x_{i}\left(l\right)}{x_{i}\left(1\right)},l=1,2,\cdots ,n$$

Then *X_i_D* is the initialing operator’s image. The initialing operator solves the problem of different dimension and it is the basis of subsequent grey system analysis.

### 3.2. Grey Incidence Analysis

GIA is a very important content in grey system theory. It studies the relevance between the system’s behavior data sequence and the relevant factors data sequence. GIA mainly includes Deng’s degree of incidence, relative degree of incidence, absolute degree of incidence and so on [23]. In this paper, the absolute degree of incidence (*ε*) was used to analyze the data sequence.

Let *Y*_0_ = (*y*_0_(*i*), *i* = 1, 2, …, *m*) be the system’s behavior data sequence, *Y*_1_ = (*y*_1_(*j*), *j* = 1, 2, …, *m*) to be the relevant factor data sequence. The absolute degree of grey incidence is calculated as follows:

Step (1). Compute the zero-stating point images.

(4) $$y_{0}^{\prime 0}\left(j\right)=y_{0}^{\prime }\left(j\right)-y_{0}^{\prime }\left(1\right),j=1,2,\cdots ,m$$

(5) $$y_{1}^{\prime 0}\left(j\right)=y_{1}^{\prime }\left(j\right)-y_{1}^{\prime }\left(1\right),j=1,2,\cdots ,m$$

Step (2). Calculate the relate parameters of the absolute degree of incidence.

Based on Grey System Theory, parameter *s_j_* is defined as

(6) $$s_{0}=\int_{1}^{m}\left(Y_{0}-y_{0}\left(1\right)\right)dt$$

(7) $$s_{1}=\int_{1}^{m}\left(Y_{1}-y_{1}\left(1\right)\right)dt$$

(8) $$s_{0}-s_{1}=\int_{1}^{m}\left(Y_{0}-Y_{1}\right)dt$$

Step (3). Compute the absolute degree of incidence *ε*.

(9) $$\varepsilon_{01}=\frac{1+\left|s_{0}\right|+\left|s_{1}\right|}{1+\left|s_{0}\right|+\left|s_{1}\right|+\left|s_{0}-s_{1}\right|}$$

According to the grey system theory, the value of the relevant degree is limited to (0,1], the closer the value is to 1, the stronger the relevance. Using Equations (4)–(9), the absolute degree of grey incidence of two groups data sequences can be obtained.

### 3.3. Grey Prediction Model

The grey system prediction model is to extract effective information from the known data, to achieve the correct description of the system’s evolution law, and thus generates quantitative predict the future of the system [23]. This paper uses multiple relevant factor model GM(0,N) to predict and analyze the data system.

Suppose the system’s characteristic data sequence is *X*_1_^(0)^ = (*x*_1_^(0)^(1), *x*_1_^(0)^(2), …, *x*_1_^(0)^(*n*)), the relevant factors data sequence are *X*_2_^(0)^ = (*x*_2_^(0)^(1), *x*_2_^(0)^(2), …,*x*_2_^(0)^(*n*)), …, *X_N_*^(0)^ = (*x_N_*^(0)^(1), *x_N_*^(0)^(2), …,*x_N_*^(0)^(*n*)). The generation process of the GM(0,N) model is as follows:

Step (1). Generate the original data sequence into an accumulation data sequence.

(10) $$x_{i}^{\left(1\right)}\left(l\right)=\sum_{l=1}^{l}x_{i}^{\left(0\right)}\left(l\right),l=1,2,\cdots ,n$$

Step (2). Build the cumulative data sequence matrix.

(11) $$X=\left[\begin{array}{ccccc} x_{2}^{\left(1\right)}\left(2\right) & x_{3}^{\left(1\right)}\left(2\right) & \cdots & x_{N}^{\left(1\right)}\left(2\right) & 1\\ x_{2}^{\left(1\right)}\left(3\right) & x_{3}^{\left(1\right)}\left(3\right) & \cdots & x_{N}^{\left(1\right)}\left(3\right) & 1\\ \vdots & \vdots & \ddots & \vdots & \vdots \\ x_{2}^{\left(1\right)}\left(n\right) & x_{3}^{\left(1\right)}\left(n\right) & \cdots & x_{N}^{\left(1\right)}\left(n\right) & 1 \end{array}\right]$$

(12) $$B=\left[\begin{array}{c} x_{1}^{\left(1\right)}\left(2\right)\\ x_{1}^{\left(1\right)}\left(3\right)\\ \vdots \\ x_{1}^{\left(1\right)}\left(n\right) \end{array}\right]$$

Step (3). Establish the least square estimation equation.

(13) $$\hat{b}=\left(b_{2},b_{3},\cdots ,b_{N},a\right)={\left(B^{T}B\right)}^{-1}B^{T}X$$

Step (4). Generate the predictive equation of the GM(0,N) model.

(14) $$x_{1}^{\left(1\right)}=b_{2}x_{2}^{\left(1\right)}\left(l\right)+b_{3}x_{3}^{\left(1\right)}\left(l\right)+\cdots +b_{N}x_{N}^{\left(1\right)}\left(l\right)+a$$

The GM(0,N) model can be established by using Equations (10)–(14) to complete the predictive analysis of the system.

## 4. Experimental Procedure

The slider was made of Cr12MoV hardness die steel (700 HV hardness), and the surface topography of the slider was processed through a VL-1000E three-axis CNC vertical milling machine by a 20 mm diameter ball-end milling cutter. The slider was a cuboid with the surface topography, and its width was 7 mm. The flat plate was a cuboid with the width of 16 mm, and a circular hole with a 3 mm diameter was provided at each end of the flat plate to be fixed on the working table of the friction and wear tester. In the experiment, two groups slider surface topography with the same length and width size and the different offset distances was machined. The specific parameters are shown in Table 3.

**Figure 5:**
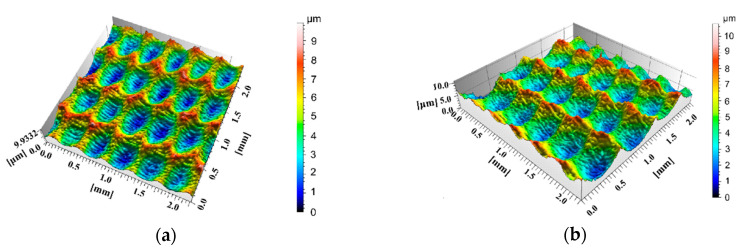
3D schematic diagram of the two groups sliders surface topography in the experiment: (**a**) Group F, the topography offset distance is 0.17 mm; (**b**) Group G, the topography offset distance is 0.08 mm.

The surface texture roughness parameters were detected by a Talysurf CCI non-contact 3D white light interference microscope. The filtering technology of the surface topography profilometer adopts the ISO/TS 16610 standard. The basic parameters are: measurement mode is coherence correlation interferometry, three position turret objectives mount, magnification power of the objective lens is 20×, work distance between sample and lens is 4.7 mm, the optical resolution (*X*,*Y*) is 0.4–0.6 μm, the vertical resolution is 0.01 nm, the noise floor is less than 0.08 nm, maximum data points are 1,048,576, the size of the measurement analysis area is 2.2 mm × 2.2 mm, and the meaning of the surface texture roughness parameters during the detection process complies with the ISO 25178-2 standard. The measured 3D surface topography of the workpiece in the experiment was shown in Figure 5.

The surface texture roughness parameters of the workpiece before sliding wear is shown in Table 4.

**Figure 6:**
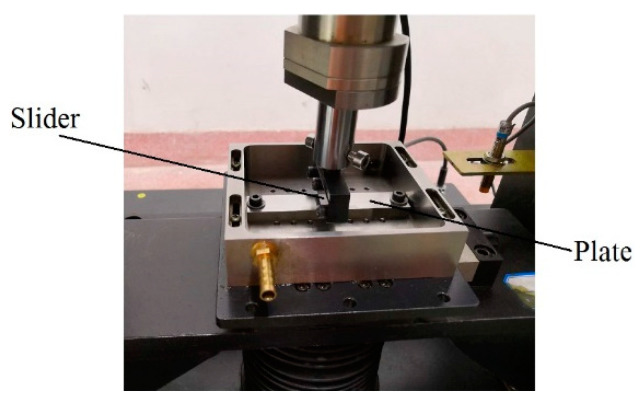
Sliding tribotester of reciprocating type.

The wear test of the slider was carried out by a reciprocating sliding tribotester under dry friction conditions. The normal load in the experiment acts on the slider, and the load sensors located directly above the slider were used to feedback the normal load. In the sliding friction experiments, the single sliding distance was 30 mm, the normal load was 100 N, and the frequency was 5 Hz. The sliding friction test equipment is shown in Figure 6.

## 5. Results and Discussion

### 5.1. Surface Texture Roughness Parameters

Surface texture roughness parameters are the most commonly used way of characterizing surface topography. To more accurately study the basic characteristics of ball-end milling surface topography with different topography offset distance, this paper selects a set of surface texture roughness parameters (*S_z_*, *S_p_*, *S_v_*, *S_q_*, *S_a_*, *S_ku_*, *S_sk_*, and *S_xp_*) to characterize and analyze the surface topography, and the meaning of surface texture roughness parameters is defined by the ISO-25178-2 standard [24]. In this paper, extract the different surface topography point cloud data, and then the surface texture roughness parameters were calculated according to the ISO-25178-2 standard. The specific data are shown in Table 5.

**Figure 7:**
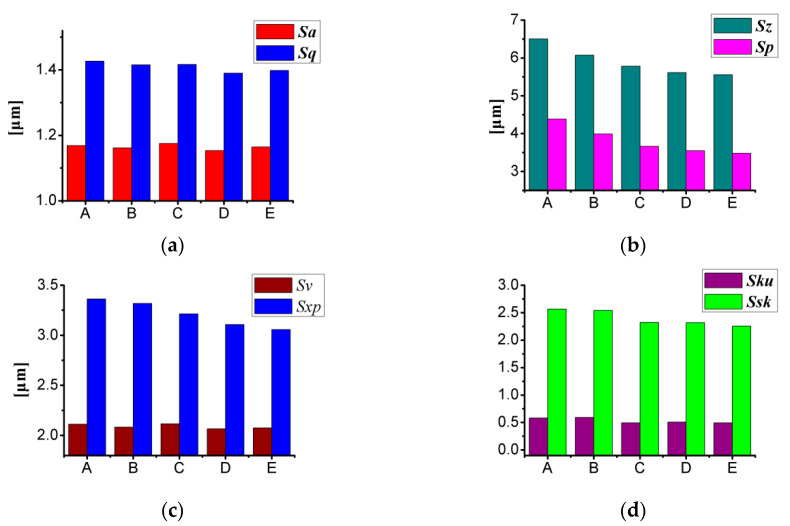
Changing characteristics of surface texture roughness parameters of surface topography with different offset distance: (**a**) *S_a_* and *S_q_*; (**b**) *S_z_* and *S_p_*; (**c**) *S_v_* and *S_xp_*; (**d**) *S_ku_* and *S_sk_*.

It can be seen from Figure 2 and Table 5 that with the change of the offset distance, the surface topography has changed, the surface texture roughness parameters also have changed. The change characteristics of the surface texture roughness parameter of the surface topography with different offset distances are shown in Figure 7.

*S_a_* is the arithmetical mean height of the surface topography, and *S_q_* is the root mean square height. It can be seen from Figure 7a that with the increase of the offset distance, both *S*_a_ and *S*_q_ have oscillating changes, and the change trend of *S_a_* and *S_q_* was consistent.

*S_z_* is the maximum height of the topography within the evaluation area, *S_p_* is the largest peak, and *S_v_* is the maximum pit height. *S_z_*, *S_p_*, and *S_v_* have a strong correlation with each other, that is, *S_z_* = *S_p_* + *S_v_*. *S_xp_* is the peak extreme height, and it is the height difference between the areal material ratio values 2.5% and 50%. It can be seen from Figure 7b,c that with the offset distance increases, *S_z_*, *S_p_*, and *S_xp_* gradually decrease, while *S_v_* show an oscillating changes characteristic similar to *S_a_*. 

*S_ku_* is defined as the kurtosis, and *S_sk_* is defined as the skewness [14]. It can be seen from Figure 7d that the *S_ku_* value gradually decreases with the surface topography from quadrilateral pits to axisymmetric hexagonal pits, and the surface topography was closer to the axisymmetric hexagon, the smaller the value of *S_ku_*. That is, with the topography offset distance gradually increase, the low-peak characteristics of the surface topography were becoming more and more obvious. *S_sk_* shows oscillating change characteristics. As the offset distance increases, *S_z_*, *S_p_*, and *S_xp_* gradually decrease, the change in *S_sk_* could be caused by the change in the pit zone.

### 5.2. Wear Simulation Analysis

**Figure 8:**
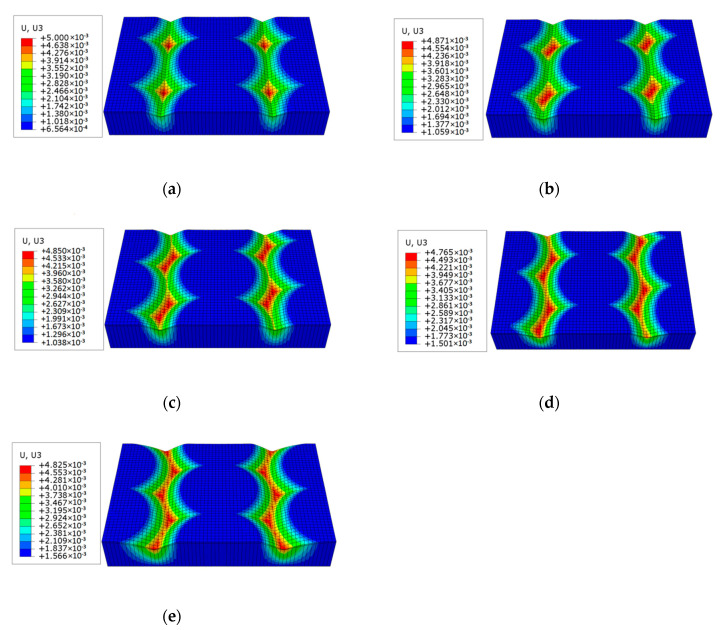
Deformation diagram of surface wear of five groups slider: (**a**) Group A, (**b**) Group B, (**c**) Group C, (**d**) Group D, (**e**) Group E. (mm).

According to the Hertz theory and Archard wear model, there is a certain contact zone between the slider and the plate before sliding wear begins. As the sliding distance increases, the contact area gradually becomes larger and the wear amount also gradually increases. In order to reflect the wear characteristics of surfaces topography, when the sliding distance is 750 m, the schematic diagrams of the sliders surface wear deformation with different surface topography was extracted. To describe the wear deformation characteristic more clearly, the wear deformation scale factor was set to 20 in the numerical simulation, and the wear deformation diagram of the five groups slider contact surface is shown in Figure 8.

It can be seen from Figure 8 that, after sliding wear, the shapes of the surface wear deformation zones of the five groups slider were not the same. As the increase of the topography offset distance, the wear deformation zones also shift. With the increase of the topography offset distance, the severely worn area, that is the red area in Figure 8, gradually increases. Due to the influence of the topography offset distance, the wear amounts of the five groups of contact surface were also different.

When the external normal load is the same, due to the different contact area, and the contact pressure is not the same, resulting in different wear amount. The wear amount of the five groups slider in the simulation analysis was indeed not the same. In order to better characterize the wear characteristics, let ∆*V_s_* be the wear amount of per unit sliding distance, then

(15) $$\Delta V_{s}=\frac{V}{s}$$

The specific data of the wear characteristics is shown in Table 6. In this paper, the wear amount refers to the wear volume.

It can be known from the Archard wear model that the wear amount is proportional to the sliding distance. To accurately characterize the wear characteristics, and this article takes ∆*V_s_* as the research object for analysis. 

Comparing the ∆*V_s_* of the five groups contact surface, it can be seen that the mutual relationship was ∆*V_s_*(B) < ∆*V_s_*(D) < ∆*V_s_*(A) < ∆*V_s_*(C) < ∆*V_s_*(E), that is, Group B has the smallest ∆*V_s_* value and the best wear resistance, while Group E has the largest ∆*V_s_* value and the worst wear resistance. Therefore, it can be seen that there was no direct relevance between wear resistance and surface shape.

From the above analysis, under the same conditions, the wear resistance of different surface was not the same. The surface texture roughness parameter is the most intuitive representation of the surface topography. For exploring the reasons that different surfaces have different wear resistance, it is necessary to correlate the surface roughness parameter with the wear characteristics.

### 5.3. Grey Incidence Analysis

During the wear process, the surface texture roughness parameters and wear characteristic parameters cannot be obtained online at any time, so the data amount that can be obtained was so small. In reciprocating sliding wear systems, its data characteristics are consistent with the research object of the grey system theory. For exploring the relationship between the Δ*V_s_* and surface texture roughness parameters, the grey incidence analysis theory is employed to analyze its relevance.

Since the dimension between Δ*V_s_* and the surface texture roughness parameters was not the same, first use Equation (3) to calculate the initialing operator for Δ*V_s_*, *S_z_*, *S_p_*, *S_v_*, *S_q_*, *S_a_*, *S_ku_*, *S_sk_*, and *S_xp_* of the five groups sliders surface topography. For different contact surfaces, we are mainly concerned about the relevance of the relative change rates between the Δ*V_s_* and the surface texture roughness parameters. Therefore, it is reasonable to use the initialing operator to calculate the relevant data. The calculation results are shown in Table 7.

Based on the data in Table 7, using Equations (4)–(9), the absolute degree of grey incidence *γ* between Δ*V_s_* and surface texture roughness parameters can be calculated. The specific values are shown in Table 8.

It can be seen from Table 8 that the absolute degree of grey incidence *ε* between Δ*V_s_* and surface texture roughness parameters, the larger the value of *ε*, the stronger the relevance. The relevance between Δ*V_s_* and surface texture roughness parameters can be clearly quantified as reflected in Table 8. The relevance between areal roughness parameters and Δ*V_s_* from strong to weak was *S_a_*, *S_v_*, *S_q_*, *S_xp_*, *S_ku_*, *S_sk_*, *S_z_*, and *S_p_*.

In addition to the high relevance between Δ*V_s_* and the overall topography parameters *S_a_* and *S_q_*, it also has a strong relevance with *S_v_*, but its relevance with height parameters, especially the peak height *S_p_*, was slightly weaker. The main reason was the unique pit shape of the surface topography. The surface is a polygonal pit topography with a certain curvature, so the wear resistance is closely related to the depth of the pit.

### 5.4. Grey Prediction Model

The surface wear resistance is closely related to the surface texture roughness parameter. Therefore, it is very important to explore the relationship between the wear resistance and the surface texture roughness parameters. Wear resistance is affected by the overall characteristics of the surface topography, a single surface texture roughness parameter cannot accurately reflect the wear resistance.

To more accurately evaluate the wear resistance of the contact surface, set the critical value of the absolute degree of grey incidence *δ* to 0.8. The surface texture roughness parameter satisfying the condition *γ* ≥ *δ* was selected as the relevant factor data sequence, and the GM(0,N) model was used to analyze it. The surface texture roughness parameters that meet the conditions were *S_a_*, *S_v_*, *S_q_*, *S_xp_*, *S_ku_*, and *S_sk_*. On this basis, a GM(0,6) model for predicting Δ*V_s_* can be established.

According to the data in Table 5 and Table 6, and using Equations (10)–(14), based on the surface texture roughness parameters, a GM(0,6) prediction model of Δ*V_s_* was established.

(16) $$\begin{array}{l} \Delta V_{s}^{\left(1\right)}=0.1217S_{a}^{\left(1\right)}+0.1529S_{v}^{\left(1\right)}+0.1314S_{q}^{\left(1\right)}-0.0790S_{xp}^{\left(1\right)}+0.1469S_{ku}^{\left(1\right)}\\ \;+0.0895S_{sk}^{\left(1\right)}-0.0553 \end{array}$$

The predicted result of the Δ*V_s_* can be obtained using the GM(0,6) model, as shown in Table 9.

It can be seen from Table 8 that the average relative errors were 0.0544%, so it is feasible to select *S_a_*, *S_v_*, *S_q_*, *S_xp_*, *S_ku_*, and *S_sk_* as relevant factors to establish a predictive model. 

In this paper, the grey system theory was used to analyze the relevance between the wear characteristic parameter Δ*V_s_* and the surface texture roughness parameters, and obtains the quantified value of the relevance. Based on this, six parameters *S_a_*, *S_v_*, *S_q_*, *S_xp_*, *S_ku_*, and *S_sk_* were selected as relevant factors, and the Δ*V_s_* was predicted by the GM(0,6) model. The Δ*V_s_* in this paper was obtained under the conditions of specific loads, topography curvature radius, topography sizes, workpiece size, etc., and the GM(0,6) model was also established under this condition.

The different surface topography would produce different surface texture roughness parameters, resulting in different wear characteristics parameter Δ*V_s_* during sliding wear. Through analysis and research, we can found that the surface wear resistance was mainly closely related to *S_a_*, *S_v_*, *S_q_*, *S_xp_*, *S_ku_*, and *S_sk_* of the surface texture roughness parameters, therefore, the surface wear resistance can be compared and evaluated based on these six surface texture roughness parameters.

The research objective of this paper is the surface topography after ball-end milling. Due to the unique cutter–workpiece contact form during the machining process, the surface topography has a pit structure. Because the wear amount of the workpiece with the different surface is closely related to the material of the friction pair, workpiece size, the sliding distance, the normal load, the size and the curvature of the surface topography, and other factors. Moreover, in the machining process of the surface topography, due to the influence of tool wear, vibration, machine tool accuracy, and other factors, the actual processed surface topography may be not exactly the same as the ideal surface topography in the simulation. However, this does not affect the accuracy of relevant analysis between the wear characteristics of the ball-end milling surface and the surface texture roughness parameters. When external conditions change, due to the complexity of wear changes, the actual value of ∆*V*_s_ may be different from the value obtained in the simulation analysis, but this will not affect the accuracy of the prediction and analysis of the different surfaces wear resistance under the same conditions. In other words, even if the external conditions have changed, it is still accurate and feasible to use the GM(0,6) model to determine the wear resistance of the different surface.

## 6. Experimental Results

According to Table 5 and Equation (16), the prediction results of the wear characteristic parameter ∆*V_s_* of the ball-end milling surface can be obtained. The ∆*V_s_* prediction value of the F group was 1.1467, and the value of the G group was 1.2388. That is, ∆*V_s_* value of F group was smaller, which shows that, under the same conditions, its wear amount was smaller and wear resistance was better.

After the friction and wear experiment, an electronic balance with an accuracy of 1 × 10^−4^ g was used to measure the wear amount on two groups of the workpieces with different surface topography. The specific data are shown in Table 10.

The density of material Cr12MoV is 7700 kg/mm^3^. Therefore, ∆*V*_s_ is expressed as

(17) $$\Delta V_{s}=\frac{m_{0}-m_{1}}{\rho s}$$

Comparing the ∆*V_s_* values of the two groups workpieces in Table 10, it can be seen that the ∆*V_s_* value of the F group was smaller than that of the G group, indicating that the surface wear resistance of the F group was better, which is consistent with the predicted result, and it also illustrates the correctness of the GM(0,6) prediction model was established in this paper.

## 7. Conclusions

This paper takes the ball-end milling surface topography of the hardened die steel material Cr12MoV as the object, and the relationship between the surface texture roughness parameters and the wear resistance was studied. The study’s general conclusions are as follows:

(1) Under dry friction conditions, the wear resistance was not directly related to the topography offset distance and the shape of the surface topography, but it was closely related to the surface texture roughness parameters.
(2) With the increase of the offset distance, the surface topography shape gradually changes from a quadrilateral pit to an axisymmetric hexagonal pit. The value of *S_z_*, *S_p_*, *S_xp_*, and *S_ku_* gradually becomes smaller, while the value of *S_a_*, *S_q_*, *S_v_*, and *S_sk_* shows an oscillating changes characteristics.
(3) Quantitative analysis of the relevance between the surfaces wear resistance and the surface texture roughness parameters under dry friction conditions, and found that there was a strong relevance between *S_a_*, *S_v_*, *S_q_*, *S_xp_*, *S_ku_*, and *S_sk_* and the wear resistance, that is, the surface wear resistance can be accurately characterized by the six surface texture roughness parameters.
(4) A zeroth order six variables grey model, GM(0,6), was established for prediction the wear characteristic parameter Δ*V_s_*, which provided a new idea for the prediction and judgment of the wear resistance of different surface topography.

## Figures and Tables

**Table 1:** Surface topography parameters of the slider in the simulation.

Groups	A	B	C	D	E
Topography offset distance (*d*/mm)	0	0.05	0.1	0.15	0.2
Slider height (*h_s_*/mm)	0.206502	0.206075	0.205783	0.205613	0.205557
Pit height (*h_p_*/μm)	6.5021	6.0750	5.7829	5.6129	5.5571

**Table 2:** Performance parameters of Cr12MoV hardened die steel in the simulation.

Material	Elastic Modulus (GPa)	Poisson’s Ratio	Density (kg/m^3^)
Cr12MoV	218	0.28	7700

**Table 3:** Surface topography size parameters of two groups workpiece.

Groups	Length (*L*/mm)	Width (*W*/mm)	Pit Height (*h_p_*/μm)	Topography Offset Distance (*d*/mm)
F	0.6	0.4	9.9332	0.17
G	0.6	0.4	10.7779	0.08

**Table 4:** Surface texture roughness parameters of the two groups workpiece before sliding wear.

Groups	*S_a_* (μm)	*S_q_* (μm)	*S_Z_* (μm)	*S_p_* (μm)	*S_v_* (μm)	*S_sk_*	*S_ku_*	*S_xp_* (μm)
F	1.4275	1.7222	9.9332	5.4729	4.4603	0.0361	2.2774	3.1434
G	1.3283	1.6267	10.7779	11.4509	4.7049	0.2559	2.4257	2.6645

**Table 5:** Surface texture roughness parameters of surface topography with different offset distance.

Groups	*S_z_* (μm)	*S_p_* (μm)	*S_v_* (μm)	*S_q_* (μm)	*S_a_* (μm)	*S_ku_*	*S_sk_*	*S_xp_* (μm)
A	6.5021	4.3890	2.1131	1.4267	1.1693	2.5658	0.5831	3.3631
B	6.0750	3.9916	2.0834	1.4152	1.1619	2.5428	0.5953	3.3185
C	5.7829	3.6669	2.1160	1.4164	1.1755	2.3241	0.4927	3.2143
D	5.6129	3.5459	2.0670	1.3897	1.1534	2.3205	0.5074	3.1079
E	5.5571	3.4810	2.0761	1.3984	1.1648	2.2578	0.4952	3.0575

**Table 6:** Wear amount of five groups slider contact surfaces when sliding distances was 750 m.

Groups	A	B	C	D	E
*V* (×10^−3^/mm^3^)	0.589303	0.589569	0.588276	0.584946	0.583111
∆*V_s_* (×10^−3^/μm^2^)	0.7857	0.7861	0.7844	0.7799	0.7775

**Table 7:** Relevant parameters values after the initialing operator.

Groups	Δ*V_s_*	*S_z_*	*S_p_*	*S_v_*	*S_q_*	*S_a_*	*S_ku_*	*S_sk_*	*S_xp_*
A	1	1	1	1	1	1	1	1	1
B	1.0005	0.9343	0.9095	0.9859	0.9919	0.9937	0.9910	1.0209	0.9867
C	0.9983	0.8894	0.8355	1.0014	0.9928	1.0053	0.9058	0.8450	0.9558
D	0.9926	0.8632	0.8079	0.9782	0.9741	0.9864	0.9044	0.8702	0.9241
E	0.9895	0.8547	0.7931	0.9825	0.9802	0.9962	0.8800	0.8493	0.9091

**Table 8:** Absolute degree of grey incidence *ε* between Δ*V_s_* and surface texture roughness parameters.

Parameters	*S_Z_*	*S_p_*	*S_v_*	*S_q_*	*S_a_*	*S_ku_*	*S_sk_*	*S_xp_*
*γ*	0.7901	0.7446	0.9729	0.9662	0.9974	0.8386	0.8061	0.8785

**Table 9:** Prediction results and errors of the GM (0,6) model.

Groups	Real Data (μm^2^)	Simulated Value (Δ*V_s_*^(1)^) (μm^2^)	Simulated Value (Δ*V_s_*^(0)^) (μm^2^)	Errors (μm^2^)	Relative Errors (%)
A	0.7857	0.7857	0.7857	0	0
B	0.7861	1.5716	0.7859	−0.0002	0.0254
C	0.7844	2.3558	0.7840	−0.0004	0.0510
D	0.7799	3.1356	0.7794	−0.0005	0.0641
E	0.7775	3.9130	0.7769	−0.0006	0.0772

**Table 10:** Experimental data of two groups workpieces after sliding wear.

Groups	*m*_0_ (g)	*m*_1_ (g)	*T* (s)	*s* (mm)	Δ*V_s_* (μm^2^)
F	62.5750	62.5492	598	89700	37.3540
G	62.4101	62.3825	598	89700	39.9600

*T*—sliding wear time; *m*_0_—initial mass of the workpiece; *s*—total sliding distance; *m*_1_—mass after wear; *ρ*—density of workpiece material.

## Nomenclature

*W*	Pit width
*L*	Pit length
*d*	Topography offset distance
*h_s_*	Slider height
*h_p_*	Pit height
*S_a_*	Arithmetical mean height
*S_q_*	Root mean square height
*S_z_*	Largest height
*S_p_*	Largest peak height
*S_v_*	Largest pit height
*S_sk_*	Skewness
*S_ku_*	Kurtosis
*S_xp_*	Difference in height between the 2.5% and 50% material ratio
